# Simultaneous bilateral reverse total shoulder arthroplasty for bilateral seizure-induced posterior shoulder fracture-dislocation complicated by severe postoperative anemia and hemorrhagic shock: a case report

**DOI:** 10.1016/j.xrrt.2024.06.001

**Published:** 2024-06-18

**Authors:** Erica Lante, Geoffroi Lallemand

**Affiliations:** a Department of Orthopedic Surgery and Traumatology, Centre Hospitalier Universitaire Vaudois (CHUV), Lausanne, Switzerland; b Department of Orthopedic Surgery and Traumatology, Riviera Chablais Hospital, Rennaz, Switzerland

**Keywords:** Bilateral posterior shoulder fracture-dislocation, Seizure-induced posterior shoulder fracture-dislocation, Bilateral reverse total shoulder arthroplasty, Simultaneous bilateral shoulder arthroplasty, Postoperative anemia, Hemorrhagic shock

Although proximal humerus fractures are the third most frequent fractures in the elderly patient after femoral neck fractures and distal radius fractures,^21^ fracture-dislocations are rare.^6^ Posterior fractures-dislocation are even rarer, amounting to 0.9% of the 1500 cases reported by Neer,^19^^,^^27^ and are associated almost exclusively with episodes of epilepsy: bilateral shoulder posterior fracture-luxation has been called “Triple-e syndrome” because it can only occur in case of extreme trauma, epilepsy or electrocution.^3^ Bilateral shoulder fracture-dislocations are so exceptional that they are only found in the literature in the form of case reports or case series.^1^^,^^16^^,^^23^^,^^24^^,^^26^, ^27^, ^28^ The treatment options for posterior shoulder fracture-dislocations, whether unilateral or bilateral, are manifold. Among them, proximal humerus osteosynthesis is the treatment of choice for young patients with good bone quality and high functional demand, but it is a treatment with difficultly predictable results and a high complication rate in the elderly patient. For this reason, with the progressive ageing of the population, surgical treatment with shoulder arthroplasties (hemiarthroplasty or total reverse shoulder arthroplasty) is increasingly used, especially with regard to total reverse shoulder arthroplasty which, compared to anatomical hemiarthroplasty, has functional results that are more predictable and independent of rotator cuff integrity. In the specific case of bilateral shoulder fracture-dislocations, another treatment described is the osteosynthesis of the proximal humerus using an autologous graft from the contralateral shoulder on one side, and a prosthesis on the other side, to increase the chances of healing the treated humerus with osteosynthesis. This case report deals with simultaneous bilateral posterior shoulder fracture-dislocation in elderly woman, managed with one-step bilateral reverse total shoulder arthroplasty and complicated by severe postoperative anemia and hemorrhagic shock. The patient was informed that data concerning his case would be submitted for publication, and he provided written consent.

## Case report

**Figure 1:**
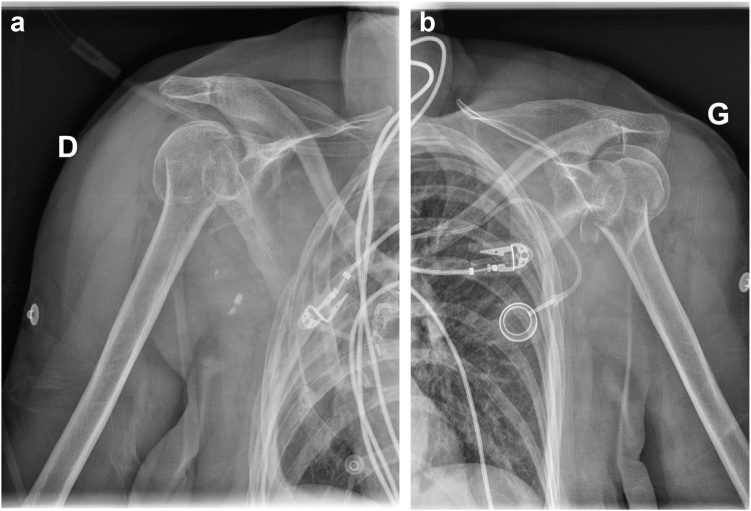
X-ray (anteroposterior) of Right (**a**) shoulder and Left (**b**) shoulder in Emergency, Department, showing bilateral posterior fracture-dislocation.

**Figure 2:**
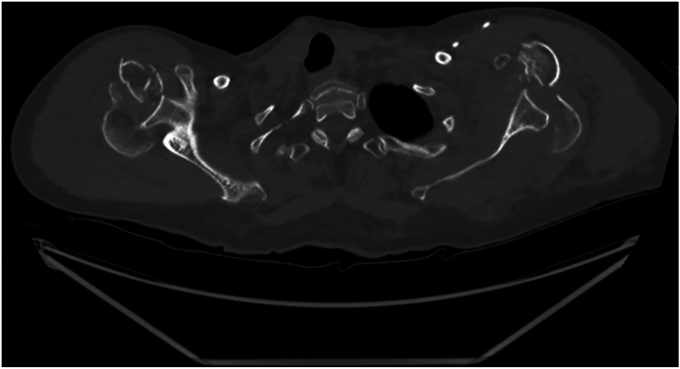
CT-scan (axial view) of bilateral shoulders performed in Emergency Department, showing bilateral posterior fracture-dislocation. *CT*, computed tomography.

**Figure 3:**
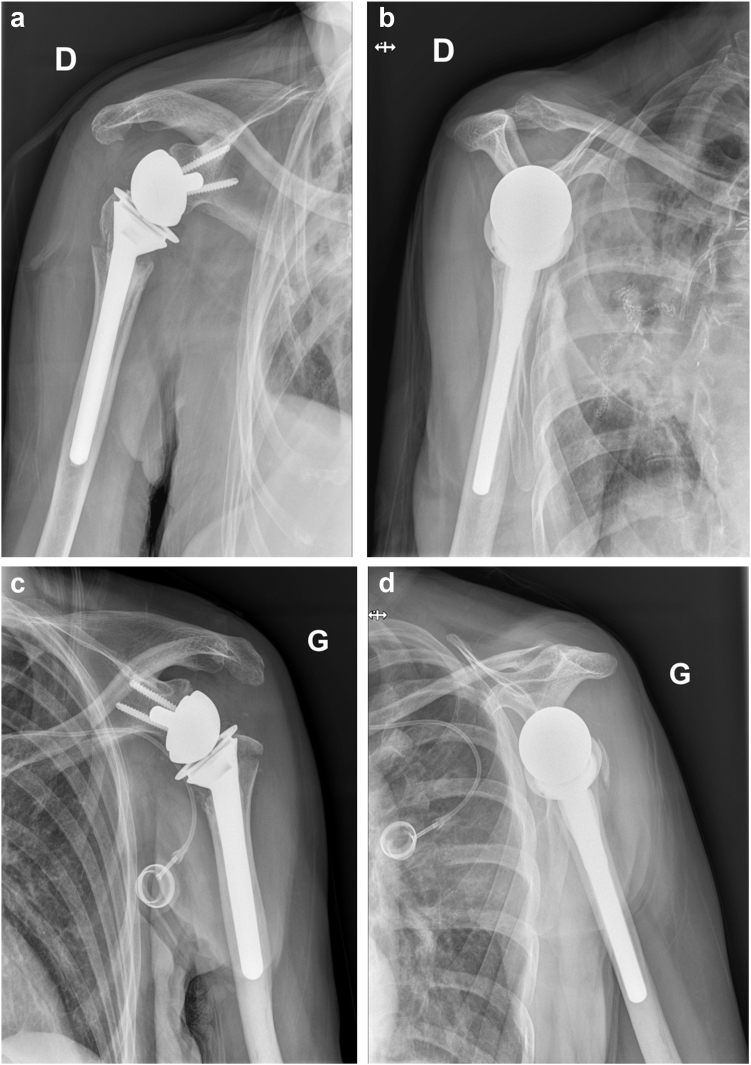
X-ray (anteroposterior, Neer views) 6 weeks postoperative of Right (**a**, **b**) shoulder and Left, (**c**, **d**) shoulder, showing correct positioning of reverse total shoulder arthroplasty on both sides.

**Figure 4:**
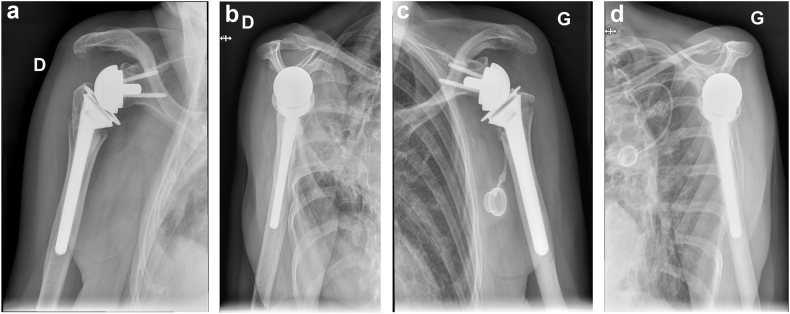
X-ray (anteroposterior, Neer views) 3 months postoperative of Right (**a, b**) shoulder and Left (**c**, **d**) shoulder, showing correct positioning of reverse total shoulder arthroplasty and healed tuberosities on both sides.

**Figure 5:**
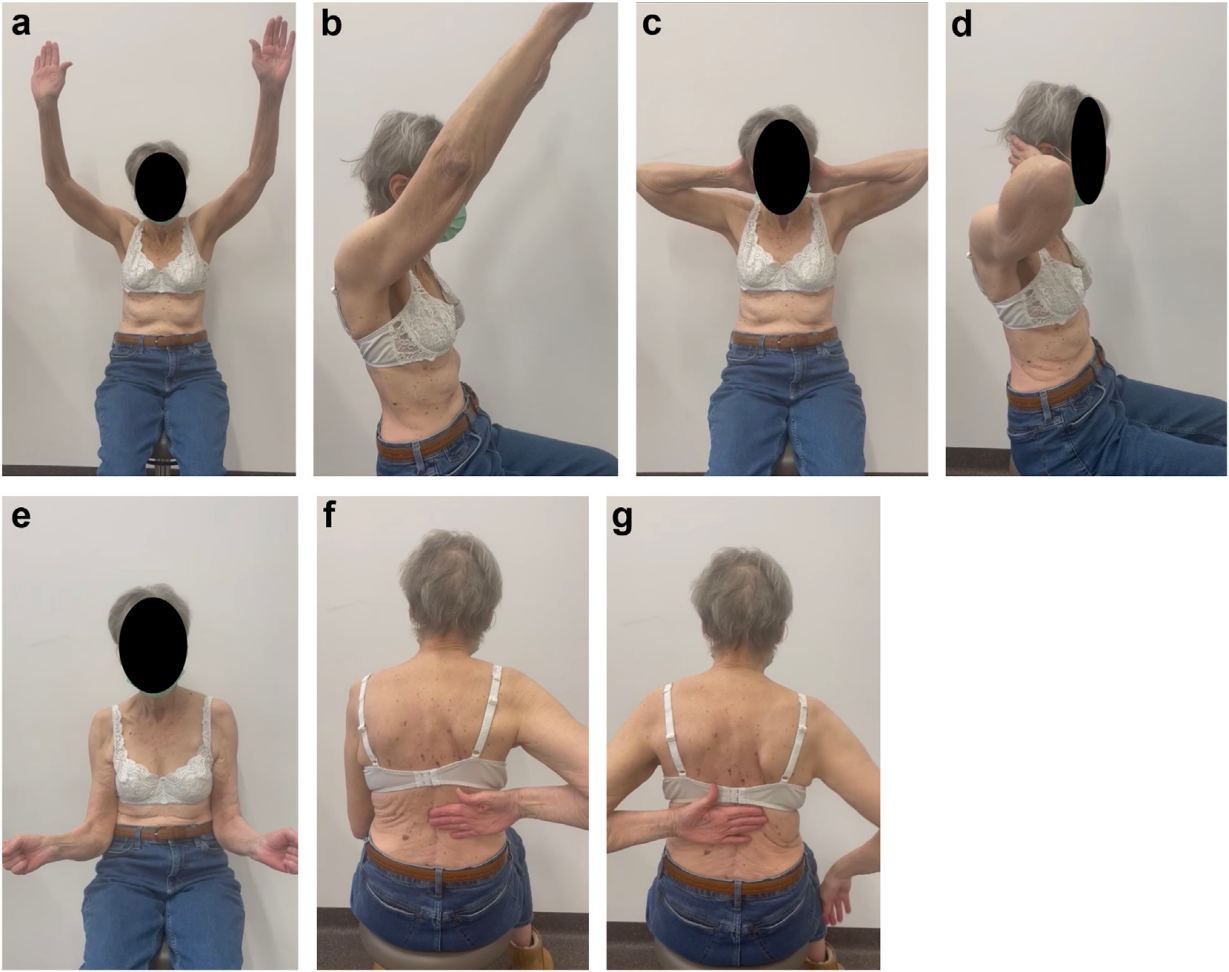
Range of motion (ROM) at 6 months postoperative ((**a** and **b**): Forward Flexion, (**c** and **d**): Abduction, (**e**) External Rotation at 0° of Abduction, (**f**) Internal Rotation of Left Shoulder at 0° of abduction, (**g**) Internal Rotation of Left Shoulder at 0° of abduction).

**Figure 6:**
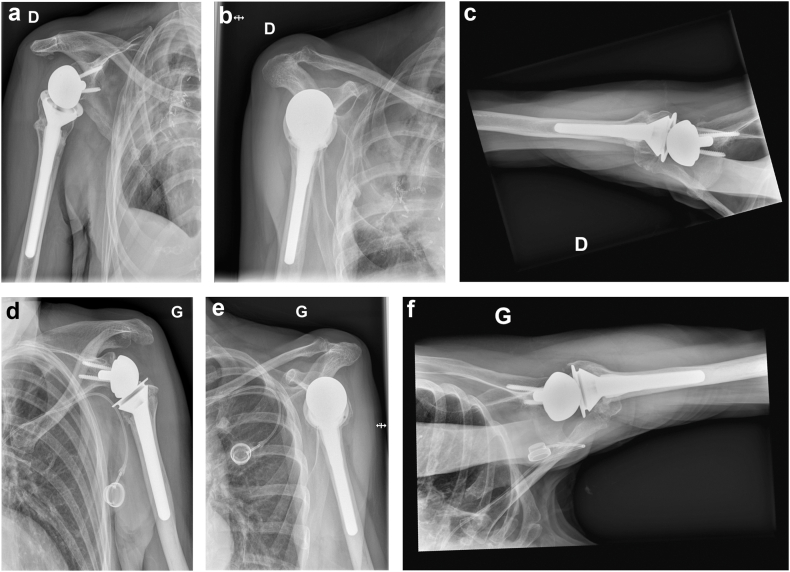
X-ray (anteroposterior, Neer, axial views) 6 months postoperative of Right (**a**-**c**), shoulder and Left (**d**-**f**) shoulder, showing correct positioning of reverse total shoulder arthroplasty and healed tuberosities on both sides.

On July 2023, a 73-year-old right-handed female patient with medical history of right lower lobar lung adenocarcinoma treated with right lung lobectomy in 2013 and chemo-radiotherapy until 2018, came to our Emergency Department (Riviera Chablais Hospital, Rennaz, Switzerland) following an episode of tonic-clonic seizure. The biological assessment revealed hyponatremia, which led the patient to be admitted to Intensive Care. The imaging evaluation (bilateral shoulder anteroposterior and Neer X-ray views and computed tomography (CT)-scan, Figs. 1, *A* and *B*, and 2) showed the presence of a bilateral shoulder fracture-posterior dislocation. The patient stayed in Intensive Care Unit for two days to determine the cause of the hyponatremia, which is still unknown. Due to the clinical history of lung carcinoma and faced with the possibility that the hyponatremia was either a paraneoplastic phenomenon due to the progression of the carcinoma or the manifestation of brain metastases, colleagues in the intensive care unit organized a comprehensive assessment to be carried out in the preoperative period. Moreover, before proceeding to surgical treatment of the bilateral shoulder fracture-dislocation, it was in any case necessary to find a precise cause of the epilepsy in order to control it and minimize the risk of recurrence. The tests carried out in Intensive Care Unit had not shown any organic causes for the hyponatremia, and the patient had not had any other episodes of tonic-clonic seizures, so our colleagues retained a possible hypovolemic origin of hyponatremia and seizures. She was then admitted to the Department of Internal Medicine and referred to our Orthopedics and Traumatology Department for management of a bilateral shoulder fracture-posterior dislocation. Physical examination showed the presence of bilateral hematoma and swelling, and a bilaterally intact function of axillary nerve. Shoulder bilateral X-Ray and chest CT-scan (Figs. 1, *A* and *B*, and 2) performed at the emergency department clarified the fracture pattern: bilateral shoulder fracture-posterior dislocation with significant comminution, type four-part according to Neer classification,^19^ 11-C3 according to AO Foundation/Orthopedic Trauma Association fracture classification.^17^ The indications, risks and benefits of surgical management were, therefore, discussed with the patient. We proposed to the patient bilateral reverse total shoulder replacement for several reasons. First among them was the patient's age, functional requirements and low activity level. Secondly, the patient had occasional shoulder pain but no limitation of daily activities prior to the convulsive episode. Both sides were operated on the same day: first her right shoulder and then her left shoulder, 14 days after injury. Since the patient was right-handed, we started with right shoulder. 1.5g of intravenous Cefuroxime was administered 30 minutes before the surgery for prophylactic purposes, and 1g of Tranexamic Acid was administered to decrease intraoperative bleeding. The surgical procedure was performed under general anesthesia and in semi-sitting position. The deltopectoral approach was used. Intraoperative assessment of the right shoulder was consistent with a 4-part fracture-dislocation according to Neer classification,^19^ with preserved insertion of the subscapularis tendon on the lesser tuberosity, preserved insertion of the supraspinatus and infraspinatus tendons on the greater tuberosity, and the teres minor tendon intact. The humeral head was dislocated posteriorly and the glenoid was intact. After releasing the fractured humeral head, the tendon of the subscapularis muscle was tagged with a FiberWire (Arthrex, Naples, FL, USA) for later repair. We then isolated the greater tuberosity and tagged the supraspinatus and infraspinatus tendons with two FiberWire (Arthrex, Naples, FL, USA). The humeral head was unimpacted and removed. The glenoid was carefully prepared and the definitive implants placed. Glenoid components included a Trabecular Metal baseplate with 15mm central plot (Zimmer Biomet, Warsaw, IN, USA), fixed with one 42mm 4.5 locking screw and one 36mm 4.5 locking screw, and a 36mm of diameter Trabecular Metal Reverse Glenoid Head (Zimmer Biomet, Warsaw, IN, USA) centered glenosphere. Humeral components were a 130mm long Trabecular Metal Reverse Humeral Stem (Zimmer Biomet, Warsaw, IN, USA), implanted with 20° of retroversion. A 36 mm of diameter and 0 mm of thickness (inlay) Trabecular Metal Reverse Poly Liner (Zimmer Biomet, Warsaw, IN, USA) was impacted. The FiberWire (Arthrex, Naples, FL, USA) sutures were used to fix the tuberosities to the humeral stem. Superior rotator cuff was fixed with transosseous sutures to proximal humerus, then the tuberosities were repaired to each other with a FiberWire (Arthrex, Naples, FL, USA) circumferential to humeral stem. For the left shoulder, the deltopectoral approach was used. Intraoperative assessment of the left shoulder was consistent with a 4-part fracture-dislocation according to Neer classification,^19^ with preserved insertion of the subscapularis tendon on the lesser tuberosity, preserved insertion of the supraspinatus and infraspinatus tendons on the greater tuberosity, and the teres minor tendon intact. The humeral head was dislocated posteriorly with associated long longitudinal metaphyseal fracture, and the glenoid was intact. It was decided to use the same implants used for the right Reverse Total Shoulder Arthroplasty for the left one. Tuberosity fixation was also performed. Intraoperative testing showed bilateral excellent stability and a range of motion of 135° of forward flexion, 90° of abduction, 45° of external rotation and 45° of internal rotation. The patient was immobilized for 6 weeks in a shoulder immobilizer in internal rotation position. Active assisted mobilization was allowed for the left shoulder from the first postoperative day, and pendulum exercise was allowed for the right shoulder from the first postoperative day. After the procedure, the patient was kept in intensive care unit for 48 hours postoperatively for surveillance purposes. The hemoglobin check in the immediate postoperative showed a normochromic normocytic anemia with hemoglobin at 92g/L (102 g/L in the immediate preoperative, with 500 ml estimated blood loss during surgery). The next morning at 8am, our patient suffered severe anemia according to National Cancer Institute definition (the hemoglobin had dropped to 79g/L and at 12 noon to 70g/L), so the patient received 1 unit of packed red blood cells. Two days after the operation, Hemoglobin was found to be 76g/L and she was then transferred to the orthopedic department. Once transferred to the orthopedic department, the patient began to experience symptoms of acute anemia, such as weakness, fatigue, pallor, tachycardia, hypotension, and shortness of breath. Hemoglobin was measured 63 g/L and the patient went into hemorrhagic shock, necessitating a transfer to intensive care unit and the transfusion of 2 unit of packed red blood cells. She stayed three days in the Intensive Care Unit, with progressive improvement of vital parameters and hemoglobin, and finally returned to the orthopedic department five days after the operation. No other source of bleeding apart from bilateral posterior shoulder fractures-dislocations and the surgery was found to be the cause for the hemorrhagic shock. In retrospect, we could have performed an injected CT scan of both shoulders looking for active bleeding. This (or other complementary tests) was not considered necessary, as the scars were calm, there was no large hematoma, and the clinical evolution was good. As the clinical evolution was favorable and the hemoglobin was 102 g/L, the patient was discharged 14 days after surgery. When the patient was discharged, the postoperative clinical examination showed the absence of axillary and suprascapular nerve lesions. Given the shoulder immobilizer on both sides, and especially on the dominant arm, the patient's age and the complications experienced postoperatively, a period of stay in a readaptation center was necessary. The patient remained in the readaptation center for 37 days, which added to the 14 days spent in hospital makes 51 days in a hospital facility away from home. Day 45 X-ray (Fig. 3, *A*-D) showed correct positioning of reverse total shoulder arthroplasty and tuberosities on both sides, and the patient stated her Subjective Shoulder Value (SSV) was 10% for right shoulder and 20% for left shoulder. Constant score was 25 for the right shoulder and 35 for the left shoulder. At physical examination right shoulder/left shoulder active forward flexion was 45°/90°, abduction was 45°/75°, external rotation 1 (ER1) was 0°/10° and internal rotation 1 (IR1) was to buttock/L4. After the six-week check-up, the patient contracted a SARS-Cov-2 infection, which was the reason why she was not able to perform all the physical therapy sessions scheduled prior to the 3-month postoperative check-up. Good clinical evolution was observed at 3 months postoperative follow-up, with SSV 60% for right shoulder and 70% for left shoulder. Constant score was 59 for right shoulder and 61 for left shoulder. At physical examination right shoulder/left shoulder active forward flexion was 90°/110°, abduction was 90°/90°, ER1 was 10°/20° and IR1 was to L1/L1, with bilateral 4/5 muscular strength according to Medical Research Council scale for resisted active forward flexion, abduction, IR1, and ER1. Rotator cuff testing was painless and force was 4/5 bilaterally. Three-month follow-up X-ray (Fig. 4, *A*-*D*) showed correct positioning of reverse total shoulder arthroplasty and healed tuberosities on both sides. At 6 months postoperative follow-up SSV was 90% for both shoulders. Constant score was 89 for both shoulders. Physical exam showed muscular force M5/M5 (bilateral normal muscular strength according to Oxford strength scale) and the following active range of motion: forward flexion 135°/135° (Fig. 5, *A* and *B*), abduction 160°/160° (Fig. 5, *C* and *D*), external rotation 45°/45° (Fig. 5, *E*) and IR1 L1/L1 (Fig. 5, *F* and *G*). SSV was 90% for both shoulders. Constant score was 89 for both shoulders. Six-month follow-up X-ray (Fig. 6, *A*-*F*) showed no postoperative complications on both sides. In view of the clinical and radiological evolution, no further controls were planned at the senior author’s consultation.

## Discussion

Posterior shoulder fracture-dislocation is a rare entity, but it is also quite typical injury for young, male patient at their first or second episode of seizure.^4^^,^^6^ We managed a bilateral shoulder fracture-dislocation in a female elderly patient with poor bone quality (deltoid tuberosity index 1.35^25^), which is not typical. In our case pure humeral osteosynthesis was not indicated for the high risk of complications, first of all humeral head necrosis because of the fracture pattern according to Hertel’s criteria^14^ (comminuted fracture of the greater and lesser tuberosities, dislocation of the humeral head without medial calcar support) and second humeral osteosynthesis failure because of the poor bone quality,^25^ nor shoulder hemiarthroplasty for the high risk of rotator cuff deficiency, complications such as tuberosities nonunion or resorption, and worst functional outcomes (forward elevation, abduction) compared to total reverse shoulder arthroplasty. For the surgical approach, we chose a deltopectoral approach. There are several reasons for this choice: first, the senior surgeon is more accustomed to the deltopectoral approach than to the superolateral approach. Our patient was a fragile patient with a bilateral fracture-dislocation, and the most logical choice was to use the most practical surgical approach for the surgeon to reduce operative time and complications. This is an a-traumatic surgical approach with a well-defined internervous and intramuscular plane.^11^ The deltoid muscle, which is the main player in shoulder mobility after reverse total shoulder arthroplasty (RTSA), is spared in this approach. In contrast, although it provides better exposure to the glenoid especially in the case of prosthesis-fracture, in the superolateral approach the origin and muscle bellies of the deltoid are violated. Since it was a poor-quality bone, if we had intraoperative fractures we could have extended the deltopectoral approach with the anterior approach to the humerus. We used a fracture designed humeral stem to improve anatomic healing and consolidation of tuberosities,^20^ and we didn’t allow passive nor assisted-active right shoulder movement for 6 weeks to allow tuberosities healing. This choice was made on the basis of the evidence that in the case of reverse total shoulder arthroplasty following a fracture the most important prognostic factor for functional outcomes (improving in internal and external rotation) is the correct positioning of the tuberosities^9^: the tuberosities were reinserted on both sides but we preferred to protect the right shoulder for 6 weeks because it was the dominant shoulder. We could have performed an autologous bone graft augmented osteosynthesis for dominant side and contralateral total reverse shoulder arthroplasty in order to improve shoulder function, as described in Uppal et al^27^ case series, but patient in their case series were on average more than 10 years younger than our patient, and we decided to perform a bilateral reverse total shoulder arthroplasty for more predictable results. However, in our case, we proposed directly to the patient a one-step bilateral reverse total shoulder arthroplasty because, as explained earlier, the functional results of a fracture prosthesis lie largely in the anatomical reduction of the tuberosities, which would have been much more difficult for the second prosthesis if this had been done at a distance from the first (especially considering that we would have waited 3 months to start getting results). In addition, according to recent literature, postfracture RTSA have better functional outcomes when operated on acutely,^2^ and recovery times are shorter than with delayed RTSA.^22^ For a 73-year-old patient, moreover, having one of the two shoulders immobilized for a long period (3 months waiting for 'surgery) would have resulted in significant atrophy of the rotator cuff muscles. Our patient suffered postoperative hemorrhagic shock, requiring the transfusion of 3 packed blood cells, and for this reason she remained hospitalized for 51 postoperative days. This finding is in line with the results of the study conducted by Gerber et al,^10^ who compared the outcomes and complications of bilateral reverse total shoulder arthroplasty for primary glenohumeral osteoarthritis implanted in a single-stage or two-stage procedure. According to their study, in fact, postoperative anemia requiring transfusion is more frequent in the group of patients with bilateral shoulder prosthesis in one time (statistically significant result). Severe anemia, although a common complication of total shoulder replacements, is a predictive factor for increasing in length of stay (in our case the patient spent 51 days in a hospital facility) and poorer 30-days outcomes^8^ and should be avoided, if possible, with bleeding control, postoperative blood test, packed red blood cells transfusion if needed. Other factors that negatively influence the length of the hospital stay are aging, female gender, the presence of preparatory anemia, congestive heart failure or renal failure.^18^ In light of the postoperative anemia, we could have used a Cell saver (Cell Saver Elite; Haemonetics, Boston, MA, USA) during surgery to perform an autotransfusion. In fact, when analyzing the literature retrospectively, our patient had all the recognized risk factors for postoperative anemia requiring postoperative transfusion of packed red cells: blood transfusion rates after total shoulder arthroplasty vary between 4.5% et 43%, and the main proven risk factors are preoperative anemia with Hb < 109 g/L (for our patient the intensive care unit assessment showed a hemoglobin of 108 g/L), and a total intraoperative blood loss (right and left shoulder together) >300 ml (for our patient estimated blood loss was 500 ml). The above two factors, together with a history of anemia, have been shown to be predictive of the need for postoperative transfusion with a sensitivity of 80.0% and specificity of 99.6%.^5^ According to another study, the indication for which a shoulder prosthesis surgery is performed makes a difference in terms of the postoperative need for transfusion: the indications most at risk are fracture-arthroplasties and revisions of arthroplasties due to infection, and since ours was a fracture prosthesis, this increases the risk of the postoperative need for transfusion. In terms of prosthesis design, reverse shoulder arthroplasties are more at risk than anatomical ones.^15^ Finally, factors negatively influencing the need to transfuse postoperatively for a shoulder prosthesis are age over 65 and female gender.^12^^,^^13^ All of the above factors are independent risk factors for the need for postoperative transfusion for a single shoulder replacement. Our patient had all or almost all of the identified factors, so it was not difficult to assume that she would almost certainly develop severe postoperative anemia. Even further, as we had done the same surgery on both shoulders at the same time, it is not surprising that the patient went into hemorrhagic shock. In spite of this, we did not reason in terms of postoperative anemia because we were misled by the fact that this was a relatively healthy patient and because of her condition of extreme discomfort dictated by the presence of the bilateral dislocation fracture and her will to get well as soon as possible. We administered 1 g of tranexamic acid to the patient, which has been shown to reduce intraoperative bleeding in RTSA, but at the same time has no effect on postoperative complications, particularly the need for transfusions.^7^ Anyway, to the best of our knowledge, postoperative anemia does not influence mid- and long-term functional outcomes. In conclusion, in our opinion, simultaneous bilateral shoulder replacement is a good option for a bilateral dislocation fracture in elderly patients, provided that due care and precautions are taken in the management of any postoperative anemia.

## Conclusion

Single-stage bilateral RTSA is a valid option for the treatment of bilateral shoulder fractures-dislocations in elderly patients, but a preoperative assessment and appropriate precautions must be included for the possible complication of postoperative anemia, and postoperative anemia risk factors should not be neglected.

## Disclaimers:

Funding: No funding was disclosed by the authors.

Conflicts of interest: The authors, their immediate families, and any research foundation with which they are affiliated have not received any financial payments or other benefits from any commercial entity related to the subject of this article.

Patient consent: The authors confirm that the patient was informed that data concerning his case would be submitted for publication, and he provided written consent.
